# Twinning rate in a sample from a Brazilian hospital with a high standard of reproductive care

**DOI:** 10.1590/S1516-31802001000600007

**Published:** 2001-11-01

**Authors:** Gloria Maria Duccini Dal Colletto, Conceição Aparecida de Mattos Segre, Bernardo Beiguelman

**Keywords:** Twinning rate, Maternal age, Gestation order, Assisted reproductive techniques, Taxa de nascimentos gemelares, Idade materna, Ordem gestacional, Fertilização assistida

## Abstract

**CONTEXT:**

Epidemiological studies on twin births have been motivated mostly by the positive correlation between twinning rate and human fertility, prematurity, low birthweight, increased risk of infant death and long term risk for morbidity.

**OBJECTIVE:**

This paper intends to estimate the incidence of multiple births in a private hospital in Brazil with a high standard of reproductive care, and to evaluate the effects of maternal age, gestation order and assisted fertilization on twinning rate.

**DESIGN:**

Retrospective analysis.

**SETTING:**

First-class tertiary private hospital, São Paulo, Brazil.

**PARTICIPANTS:**

The multiple birth rate was investigated among 7,997 deliveries from 1995 to 1998, including 7,786 singletons, 193 twins, 17 triplets and one quadruplet.

**RESULTS:**

The rates per 1,000 dizygotic and monozygotic pairs and for triplets were estimated as 19.51,4.50 and 2.13, respectively. The dizygotic and triple trates were the highest observed in Brazil up to the present day. The twinning rate among primigravidae older than 30 years was very high(45.02 per 1,000) and was due to a disproportionately high frequency of dizygotic pairs. The triple trate was also very high among the mothers of this age group (5.71 per 1,000). These facts ares trong indicators that these women were the ones most frequently submitted to assisted reproductive techniques. The mean maternal age of the studied population was about six years higher than that estimated for mothers in the general population of south eastern Brazil. Primigravidae aged under 30 years as well as multigravidae showed similar twinning rates, which were almost 20 per 1,000. Among the deliveries of multigravidae older than 30 years, an unusually high frequency of monozygotictwins was observed(7.04 per 1,000), probably as a consequence of the residual effect of long-term use of oral contraceptives.

**CONCLUSIONS:**

The dizygotic twinning rate increased from 13.51 to 28.98per 1,000 over the four years studied, with the twinning rate for primigravidae over 30 years old in 1998 being twice that observed in 1995. The mean maternal age was also high during this period, but the extremely high increase in twinning rate observed cannot be attributed solely to this variable. Assisted fertilization seems to be the most probable cause of this unusually high twinning rate.

## RESUMO

**CONTEXTO:**

Os estudos epidemiológicos de nascimentos de gêmeos têm sido motivados principalmente pela correlação positiva entre a taxa de gemelaridade e a fertilidade humana, prematuridade, baixo peso ao nascer e risco aumentado de morbimortalidade.

**OBJETIVO:**

Este trabalho pretende estimar a incidência de nascimentos múltiplos em um hospital particular brasileiro com alto padrão de cuidados reprodutivos, e avaliar os efeitos da idade materna, ordem gestacional e fertilização assistida sobre a taxa de gemelaridade.

**TIPO DE ESTUDO:**

Análise retrospectiva.

**LOCAL:**

Hospital Israelita Albert Einstein, São Paulo, Brasil.

**PARTICIPANTES:**

A taxa de nascimentos múltiplos foi estudada levando em conta 7.997 partos ocorridos entre 1995 e 1998, os quais incluíram 7.786 nascimentos de parto único, 193 gêmeos, 17 trigêmeos e um quadrigêmeo.

**RESULTADOS:**

As taxas de nascimento por 1.000 de gêmeos dizigóticos e monozigóticos e de trigêmeos foi estimada, respectivamente, como igual a 19,51, 4,50 e 2,13. As taxas de nascimento de gêmeos dizigóticos e de trigêmeos foram as mais altas no Brasil até o presente. A taxa de gemelaridade entre as primigestas acima de 30 anos foi muito alta (45,02 por mil) e decorreu de uma freqüência desproporcional de pares dizigóticos. A taxa de trigêmeos também foi muito alta nesse grupo de mães (5,71 por mil). Esses fatos são fortes indicadores que essas mulheres foram, mais freqüentemente, submetidas a técnicas de fertilização assistida. A média da idade materna da população estudada foi cerca de seis anos mais alta do que a média estimada para a população do sudeste brasileiro e mostrou uma tendência de aumento no período estudado. As primigestas com menos de 30 anos de idade e as multigestas acima de 30 anos de idade apresentaram taxas similares de gemelaridade, atingindo quase 20 por mil nascimentos. Entre os partos das mães multigestas acima de 30 anos de idade, foi observada uma alta freqüência de gêmeos monozigóticos (7,04 por mil), a qual pode ser atribuída, mais provavelmente, a efeitos residuais provocados pelo longo tempo de uso de anticoncepcionais orais.

**CONCLUSÕES:**

A taxa de gêmeos dizigóticos aumentou de 13,51 para 28,98 por mil nos quatro anos estudados, sendo que, em 1998, a taxa de gêmeos gerados por primigestas com mais de 30 anos de idade dobrou em relação a 1995. A média da idade materna também foi alta nesse período, mas o aumento exagerado da taxa de nascimentos gemelares não pode ser atribuído somente a essa variável. A fertilização assistida parece ser a causa mais provável dessa taxa de gêmeos excepcionalmente alta.

## INTRODUCTION

The causes of temporal variation in the twinning rate are not always clear.^1^ As a consequence, the study of this variation has attracted the attention of several authors who were also interested in the correlation between twinning rate and human fertility.^2,3^ Prematurity, low birth weight, increased risk of infant death and long-term risk for morbidity associated with multiple gestations have been another source of motivation for epidemiological studies on twin births.^4^

In urban populations of countries where assisted fertilization is not widespread, a decline in dizygotic twinning has been observed that was positively correlated with parity, independent of age.^5-11^ With regard to the monozygotic twinning rate, most populations have shown stability of its incidence, but a few authors have observed a slight increase in monozygotic twin births.^1,2,10-13^ The rise in monozygotic twinning might be explained as a consequence of residual effects of long term use of oral contraceptives. Such effects include depression in tubal motility and changes in the endometrial mucosa and oviduct epithelium,^11-13^ which are considered as factors favoring the birth of monozygotic twins. Thus, in animal models, delayed implantation of the embryo is associated with polyembryony in armadillos, while delayed ovulation, frequently occurring in the first cycle after withdrawal of oral con- traceptives,^14^ induces monozygotic twinning in rabbits.^15^

In Brazil, the hormone treatment on women to induce ovulation, whether followed by *in vitro* fertilization or not, is mostly performed in private clinics. Therefore, it is a reasonable supposition that women with a high standard of life attended at private hospitals would include a high proportion of those who have had assisted fertilization, which is associated with multiple births. Moreover, since women with high socioeconomic and cultural levels postpone procreation, their mean reproductive age tends to be higher than that observed in the general population. Thus, another cause influencing the increase in multiple births in these hospitals is maternal age, which is correlated with twinning, due to an increase of dizygotic pairs.^5-11^

This paper had the objective of estimating the frequency of multiple births in a first-class tertiary private hospital in Brazil.

## METHODS

The twinning rate from 1995 to 1998 was investigated at the Hospital Israelita Albert Einstein (a first-class tertiary private hospital) in São Paulo, SP, Brazil. During this period 7,997 deliveries were attended (7,786 singletons, 193 twins, 17 triplets and one quadruplet). This number included stillborn children, but excluded fetuses weighing 500 g or less at birth. Those fetuses were classified as abortions, since that fetal weight corresponds to a gestational age of between 20 and 22 weeks.^16^ The multiple birth rates referred to the number of these deliveries per 1,000, including live births and stillborn children. As the twins were not classified by any available zygosity test, the frequency of dizygotic pairs was estimated by means of the classic Weinberg rule,^17^ based on the number of unlike sex pairs.

Mean and standard deviation (SD) estimates, analyses of variance, and nonparametric tests (Mann-Whitney and Kruskal-Wallis) to compare two or more groups, were performed with the help of the SPSS 9.0 program.

## RESULT

Data on single and multiple births covering the period from 1995 to 1998, and on the rates per 1,000 dizygotic and monozygotic pairs, twin births as a whole, and triplets are shown in Table 1. Table 2 indicates the twinning rates from 1995 to 1998 among mothers grouped according to both the number of gestations (primigravidae and multigravidae) and age (less than 30 and older than 30 years), as well as the mean ages of the mothers of singletons and twins.

**Table 1:** Numbers of singletons, twins classified according to sex (MM = both males; FF = both females; MF = unlike sex pairs) and triplets, as well as the rate per 1,000 births of dizygotic (DZ), monozygotic (MZ), dizygotic plus monozygotic twins, and triplets

*YEARS*		*NUMBER OF BIRTHS*					*RATE PER 1,000 BIRTHS*		
	*SINGLETONS*	*MM*	*FF*	*MF*	*TRIPLETS*	*DZ*	*MZ*	*DZ+MZ*	*TRIPLETS*
***1995***	***1887***	***10***	***11***	***13***	***3***	***13.51***	***4.16***	***17.67***	***1.56***
1996*	***1927***	***11***	***18***	***17***	***5***	***17.19***	***6.07***	***23.26***	***2.53***
***1997***	***2069***	***24***	***13***	***18***	***2***	***16.93***	***8.94***	***25.87***	***0.94***
***1998***	***1903***	***12***	***15***	***30***	***7***	***28.98***	***0.00***	***28.98***	***3.56***
***Total***	***7786***	***57***	***57***	***78***	***17***	***19.51***	***4.50***	***24.02***	***2.13***

* Onesetofquadrupletswasborn in 1996.

**Table 2:** Twinning rate per 1,000 (TR) from 1995 to 1998 among mothers grouped according to both the number of pregnancies (primigravidae and multigravidae) and age (< 30 and ≥ 30 years). Dizygotic (DZTR) and monozygotic (MZTR) twinning rates per 1,000 observed in each group, as well as the mean age of the mothers of singletons and twins

*YEAR*		*PRIMIGRAVIDAE*				*MULTIGRAVIDAE*			
		*< 30 years*		≥ *30 years*		*< 30 years*		≥ *30 years*	
		*WOMEN*	*TR*	*WOMEN*	*TR*	*WOMEN*	*TR*	*WOMEN*	*TR*
***1995***		***415***	***14.46***	***338***	***23.67***	***316***	***15.82***	***837***	***17.92***
***1996***		***387***	***15.50***	***397***	***52.90***	***316***	***22.15***	***845***	***14.20***
***1997***		***394***	***22.84***	***426***	***44.60***	***325***	***15.38***	***979***	***22.47***
***1998***		***357***	***19.61***	***416***	***55.29***	***297***	***26.94***	***890***	***22.47***
***TOTAL***		***1553***	***18.03***	***1577***	***45.02***	***1254***	***19.94***	***3551***	***19.43***
***DZTR***		***14.17***		***44.39***		***15.95***		***12.39***	
***MZTR***		***3.86***		***0.63***		***3.99***		***7.04***	
***Mean Age in Years***	***Singletons***	***25.98 (SD 3.79)***		***33.22 (SD 2.94)***		***26.86 (SD 2.17)***		***34.01 (SD 3.13)***	
	***Twins***	***26.36 (SD 2.67)***		***33.59 (SD 3.40)***		***27.16 (SD 2.25)***		***34.20 (SD 3.32)***	

Over the analyzed period, the mean ages of the mothers of singletons, twins, and triplets were estimated as 31.12 years (SD 4.60), 31.93 years (SD 4.49), and 31.18 (SD 5.71), respectively, while the mean gestation orders were estimated as 2.02 (SD 1.15) for singletons, 1.82 (SD 1.13) for twins, and 1.19 (SD 0.40) for triplets. The distributions of the mean ages during this period were homogeneous among the mothers of twins (F_(3;189)_ = 0.70; P = 0.552) and triplets (F_(3;13)_ = 0.38; P = 0.772), but were heterogeneous among the mothers of singletons (F_(3;∞)_ = 4.29; P = 0.005) due to a rising trend in these means. Concerning the distributions of the mean gestation order of the newborns, the Kruskal-Wallis test applied to the data from 1995 to 1998 favored the hypothesis of homogeneity, since the results were 5.28 (P = 0.153) for singletons, 1.92 (P = 0.589) for twins, and 4.90 (P = 0.180) for triplets.

## DISCUSSION

The mean rates per 1,000 births calculated in Table 1 for dizygotic twins (19.51) and for triplets (2.13) are the highest described in Brazil up to the present day. These rates are respectively about 4 and 14 times higher than the estimates made for women in southeastern Brazil belonging to all social classes (4.72 per 1,000 for dizygotic twins and 0.15 per 1,000 for triplets).^11^ The annual figures in Table 1 undoubtedly show a striking increase in the dizygotic twinning rate and, as a consequence, a rise in the frequency of twins as a whole. On the contrary, the mean monozygotic twinning rate (4.5 per 1,000) was similar to that found previously in Brazil (4.1 per 1,000). ^11^

When the mothers were grouped according to both the numbers of gestations and age (Table 2), it became clear that the children born to primigravidae older than 30 years exhibited the highest twinning rate, which more than doubled after 1995. Moreover, it was observed that 53% of the total number of triplets were born to this group of mothers. The extremely high mean twinning rate observed among the children born to primigravidae older than 30 years (45.02 per 1,000) was similar to that found in Denmark among primigravidae of the same age group.^18^ This was mostly a consequence of a disproportionate increase in dizygotic pairs (44.39 per 1,000, i.e. almost 99% of the twin births from this group). This fact, together with the high frequency of triplets among them, is a strong indication that the primigravidae older than 30 years were the ones most frequently submitted to induction of ovulation, whether followed by *in vitro* fertilization or not. Otherwise, they are in agreement with the observations that fertilization treatments are associated with both decreased fecundity and increased maternal age.^19^

The very high mean twinning rate for primigravidae older than 30 years cannot be considered as responsible for the overall high twinning rate in the Hospital Israelita Albert Einstein, since the mean twinning rates indicated in Table 2 for the newborns of other groups of mothers were, in all cases, almost 20 per 1,000. This is twice as high as the twinning rate estimated for the general population of southeastern Brazil (8.8 per 1,000).^11^

The unusually high twinning rate values observed in the Hospital Israelita Albert Einstein, even after excluding primigravidae older than 30 years, may be partly attributed to age, since maternal age is positively correlated with twinning rate.^5-11^ Women attended to in the Hospital Israelita Albert Einstein are older (mothers of singletons = 31.12 years ± 4.60; mothers of twins = 31.93 years ± 4.49) than those of the general population of southeastern Brazil (mothers of singletons = 24.98 years ± 5.99; mothers of twins = 26.82 ± 6.02).^20^

However, age cannot be considered the only cause of this increase either, since according to Table 2 the mean twinning rate was also high among newborns of primigravidae younger than 30 years (18.03 per 1,000), with the proportion of dizygotic pairs still being high (14.17 per 1,000) or about 79% of the twin births. Therefore, it seems plausible to suppose that induction of ovulation, whether followed by *in vitro* fertilization or not, may also be responsible for the increase of twin births from primigravidae under 30 years of age. This hypothesis is strongly supported by the fact that 23.5% of the triplets and the only case of quadruplets were born to mothers of this age group.

Among the multigravidae older than 30 years who, of course, are rarely seeking assisted fertilization, residual effects of long-term use of oral contraceptives may be another source of elevated twin births, through the induction of increased monozygotic twin births.^11^^,^^12^ In fact, among the twins born to this group of mothers, the proportion of monozygotic pairs was very high (36%) when compared to the three other groups, with the total twinning rate and the monozygotic twinning rate being 19.43 and 7.04 per 1,000, respectively. A high frequency of monozygotic pairs among children born to mothers older than 30 years was an unexpected event, since it is known that the monozygotic twinning rate declines as maternal age increases.^21^^,^^22^

A small but significant difference (*t* = 2.422; P = 0.015) was observed when the maternal mean age for singletons (31.12 years ± 4.60) was compared to that for twins [31.93 years (SD 4.49)]. Twins also differed significantly from singletons in relation to the mean gestation order (1.82 for twins and 2.01 for singletons), the Mann-Whitney test resulting in Z = 2.97; P = 0.003. This difference is in disagreement with the classic literature that indicates the opposite, that is to say, higher gestation orders for twins than for singletons.^1,5,6,8,19,20,23,24^ However, an obvious explanation is presented if the analyzed sample includes a high frequency of women submitted to assisted reproductive techniques for inducing their first pregnancy.

The very high rate of multiple births observed in Hospital Israelita Albert Einstein, which is in fact the highest detected in Brazil, demands special attention from perinatologists. This is because in addition to perinatal morbidity-mortality risks being greater for multiple births than for singletons,^25-28^ emphasis needs to be placed on the fact that mortality among twins remains significantly higher than among singletons until the age of five years.^29^ Thus, in another Brazilian sample, when twins were compared with singletons, the likelihood of stillbirths and early neonatal deaths among twins was 1.9 and 6.5 times greater, respectively.^28^ Since, on average, twins are born three weeks earlier than singletons,^20^ while 41.2% of the triplets are born before completing 33 weeks,^4^ it is understandable that around 50% of twin births and more than 90% of triplets present low birth weight. Moreover, the frequency of very low birth weight among newborn children that are twins is several times greater than for singletons.^25,28,30,31^

## Conclusions

During the period from 1995 to 1998, the mean rates per 1,000 births of dizygotic twins (19.51) and triplets (2.13) observed in the Hospital Israelita Albert Einstein were the highest that have been described in Brazil up to the present day.

The dizygotic twinning rate increased from 13.51 to 28.98 per 1,000 over the four years studied. During this period, the twinning rate more than doubled among primigravidae over 30 years old (from 23.67 to 55.29 per 1,000).

The mean age of the women attended to in the Hospital [31.12 years (SD 4.60) for mothers of singletons, and 31.93 years (SD 4.49) for mothers of twins] was higher than that of mothers from the general population of southeastern Brazil [24.98 years (SD 5.99) for mothers of singletons, and 26.82 (SD 6.02) for mothers of twins].^20^

The striking increase in the twinning rate observed in the Hospital cannot be attributed solely to maternal age. This is because the twinning rate was also high among the newborns of primigravidae with ages under 30 years (18.03 per 1,000), with the proportion of dizygotic pairs being high and about 79% (14.17 per 1,000). Such an increase is due, most probably, to the influence of assisted fertilization, which is associated with multiple births. Due to the high prices involved in assisted fertilization in Brazil, this practice is mostly performed in private clinics that attend to women with a high standard of life, as is the case of the Hospital.

Among multigravidae older than 30 years the monozygotic twinning rate was high (7.04 per 1,000). This result could be attributed, in part, to residual effects of long-term use of oral contraceptives, although some effect of the *in vitro* fertilization cannot be discarded.
